# Fluoride Content in Infusions of Selected Teas Available on the Polish Market—An In Vitro Study

**DOI:** 10.3390/foods14142452

**Published:** 2025-07-12

**Authors:** Agata Małyszek, Ireneusz Zawiślak, Michał Kulus, Adam Watras, Julia Kensy, Agnieszka Kotela, Marzena Styczyńska, Maciej Janeczek, Jacek Matys, Maciej Dobrzyński

**Affiliations:** 1 Department of Biostructure and Animal Physiology, Wroclaw University of Environmental and Life Sciences, Kożuchowska1, 51-631 Wroclaw, Poland; maciej.janeczek@upwr.edu.pl; 2 Department of Human Nutrition, Faculty of Biotechnology and Food Science, Wroclaw University of Environmental and Life Sciences, Józefa Chełmońskiego 37, 51-630 Wroclaw, Poland; ireneusz.zawislak@upwr.edu.pl (I.Z.); marzena.styczynska@upwr.edu.pl (M.S.); 3 Division of Ultrastructural Research, Wroclaw Medical University, Chałubinskiego 6a, 50-368 Wroclaw, Poland; michal.kulus@umw.edu.pl; 4 Department of Pediatric Dentistry and Preclinical Dentistry, Wroclaw Medical University, Krakowska 26, 50-425 Wroclaw, Poland or a.watras@intibs.pl (A.W.); maciej.dobrzynski@umw.edu.pl (M.D.); 5 Institute of Low Temperatures and Structure Research, Polish Academy of Sciences, Okolna 2, 50-422 Wroclaw, Poland; 6 Faculty of Dentistry, Wroclaw Medical University, Krakowska 26, 50-425 Wroclaw, Poland; julia.kensy@student.umw.edu.pl; 7 Medical Center of Innovation, Wroclaw Medical University, Krakowska 26, 50-425 Wroclaw, Poland; kotela.agnieszka@gmail.com; 8 Dental Surgery Department, Wroclaw Medical University, Krakowska 26, 50-425 Wroclaw, Poland

**Keywords:** black teas, tea bags, fluoride release, mineral content, dietary intake, public health

## Abstract

This study aimed to evaluate the fluoride content and other key physicochemical properties in commercially available black tea infusions, with a focus on tea form and geographic origin, in order to assess their contribution to total dietary fluoride intake. Methods: A total of 121 black tea samples were analyzed, including 66 loose-leaf, 42 bags, and 13 pyramid-bag teas. Infusions were prepared using standardized brewing protocols. Fluoride concentrations were determined with an ion-selective electrode, while the pH, buffer capacity, titratable acidity, calcium, and inorganic phosphorus were also measured. Statistical analysis included ANOVA, Tukey post hoc tests, and Pearson correlation analysis. Results: Fluoride content varied significantly by tea form and origin. Infusion of tea bags exhibited the highest fluoride, calcium, and acidity levels, while loose-leaf teas had the lowest. Teas from Africa contained approximately twice as much fluoride as those from Central or East Asia. Significant correlations between fluoride, calcium, and phosphorus were observed, particularly in tea-bag infusions, suggesting processing influences mineral release. Conclusions: Black tea, particularly in bag form and sourced from African regions, may significantly contribute to daily fluoride intake. Given the potential to exceed recommended fluoride thresholds, especially in individuals consuming multiple cups daily or living in fluoridated areas, these findings underscore the importance of consumer awareness and possible product labeling to guide safe consumption.

## 1. Introduction

Tea, derived from the leaves of *Camellia sinensis*, is one of the most widely consumed beverages globally and is available in several primary types—green, black, white, and oolong—each distinguished by unique processing methods and chemical profiles [1,2,3]. Black tea, the most popular type on the global market, has the highest degree of oxidation, leading to the formation of theaflavins and thearubigins—polyphenolic compounds produced from the transformation of catechin during fermentation —which are largely responsible for its antioxidant, anti-inflammatory, and cardiometabolic effects [4,5].

In addition to these bioactive compounds, black tea contains flavonols, gallic acid, caffeine, L-theanine, and a range of minerals [6,7,8]. Among these constituents, fluoride (F) is of particular interest, as it naturally accumulates in tea leaves and is commonly present in both the dry leaves and brewed infusions. While fluoride provides protective benefits for dental health, excessive intake may lead to adverse effects [9,10]. Its concentration in tea varies widely depending on factors such as geographic origin, agricultural practices, processing techniques, and the form in which the tea is marketed [11]. Black tea products—including loose leaf, tea bags, instant powders, and concentrated extracts—differ in their fluoride content and release characteristics [8]. Therefore, evaluating fluoride concentrations across various black tea products is essential for better understanding their contribution to total dietary fluoride intake and potential health implications.

Fluoride is an essential micronutrient that plays a key role in dental prevention. The relationship between fluoride and human health exemplifies a dose–response effect, where insufficient intake is associated with an increased risk of tooth decay. At optimal concentrations, fluoride enhances enamel mineralization through the formation of fluorapatite, increasing the resistance of teeth to acid-induced demineralization and thereby reducing the incidence of dental caries [12,13]. The cariostatic properties of fluoride include both the remineralization of enamel and the inhibition of caries-causing microorganisms such as Streptococcus mutans, by reducing their adhesion to the tooth surface [14,15,16,17]. Conversely, excessive fluoride intake, particularly over extended periods, poses well-documented health risks. In children, chronic overexposure can lead to dental fluorosis, a condition characterized by enamel hypomineralization, often irreversible. In adults, long-term intake exceeding safe thresholds may contribute to skeletal fluorosis, which can cause joint pain, reduced mobility, increased bone fragility, and potentially systemic effects, including neurological and endocrine disorders [18,19,20,21].

Dietary sources contribute significantly to total fluoride intake, with drinking water traditionally considered the primary source in many communities, though considerable variation exists depending on geographical location, food processing methods, and individual dietary patterns [12,13]. The World Health Organisation (WHO) recommends a fluoride concentration in drinking water of between 0.5 and 1.5 mg/L, with an optimum concentration of around 1 mg/L [14]. In contrast, the U.S. National Institutes of Health (NIH) recommends an adequate intake (AI) of fluoride of 3 mg/day for adult women and 4 mg/day for adult men, while the upper intake limit (UL) is 10 mg/day for adults and 0.1 mg/kg per day for children [19,20,22,23]. The European Food Safety Authority (EFSA) makes similar recommendations, noting the risk of chronic fluorosis [15,16]. The WHO advises that total fluoride intake should not exceed 0.05 mg/kg per day from all sources, including water, food, beverages, and dental products [15]. Polish national recommendations align closely with international standards. The Polish Society of Pediatric Dentistry and other expert bodies emphasize individualized fluoride prophylaxis, particularly in children and adolescents. They recommend a daily intake of 0.05 mg/kg and strongly advise against additional systemic fluoride use without assessing total environmental and dietary exposure [17,18]. Tea, particularly black tea, has been identified as a significant dietary source of fluoride due to the plant’s ability to bioaccumulate the element. The fluoride content of black tea depends, among other factors, on the form of the tea (such as tea bags, sticks, or granules), brewing time, brewing temperature, water quality, and the age of the leaves—with older leaves typically containing more fluoride [24,25,26,27,28,29,30,31]. According to scientific studies, consuming 1 to 1.5 liters of black tea per day (approximately 4–6 cups) may provide 50–100% of the recommended AI of fluoride for adults, and, in some cases, certain varieties may even exceed the tolerable upper intake level (UL) [32,33,34]. In populations with habitual tea consumption, especially where water is also fluoridated, the risk of overexposure increases significantly [35,36]. The bioavailability of tea-derived fluoride appears comparable to that of fluoridated water, suggesting equivalent biological activity [19,37]. Understanding these exposure parameters becomes essential for risk assessment and developing appropriate dietary recommendations, particularly for vulnerable populations including children, pregnant women, and individuals residing in areas with naturally high fluoride concentrations in water supplies [22,35,36]. This becomes particularly significant in the context of public health recommendations, where the maximum daily fluoride intake is established at 3–4 mg/day for adults and 0.05 mg/kg body weight for children [20,23,38].

In view of the growing interest in tea as a potentially important source of fluoride and the lack of detailed data on its content in various commercial forms, we decided to undertake a study focused on black teas commonly available on the market. Despite existing research pointing to notable fluoride levels in tea, comprehensive comparisons across product types and regions of origin remain scarce. Our goal was to explore how tea form (e.g., bags, loose-leaf, and pyramid bags), geographical origin, fluoride concentrations, and other physicochemical properties of tea infusions, such as calcium (Ca) and phosphorus (P) content, pH, buffering capacity, and titratable acidity, thereby contributing to a comprehensive understanding of the potential reciprocal effects on fluoride bioavailability and its impact on human health. The presence of calcium and phosphorus ions, as well as the pH of the infusion, can influence the degree of fluoride dissolution and complexation, thereby modifying its bioavailability. Conversely, the acidity and buffering capacity of the infusion may affect the stability of fluoride compounds and their interactions with other components of the beverage [21,24,39,40]. This research is particularly relevant in light of the public health concerns regarding cumulative fluoride exposure from multiple sources. By providing a clearer understanding of how different tea products contribute to total fluoride intake, our findings may help guide more informed consumer choices and support the development of targeted dietary recommendations—especially for groups at higher risk of overexposure.

## 2. Materials and Methods

### 2.1. Study Groups

The study investigated 124 black tea products from various producers, purchased from retail stores in Wrocław, Poland. It focused on the following three types of tea: loose-leaf (*n* = 66), standard tea bags (*n* = 42), and pyramid bags (*n* = 13). The teas were categorized geographically into the following three regions: Africa (*n* = 16), Central Asia (*n* = 81), and East Asia (*n* = 14). The detailed characteristics of the black teas are included in Table 1.

### 2.2. Preparation of Tea Infusions

Tea bags, each weighing between 1.4 and 2.0 g, were infused with 200 mL of commercial deionized water (EUROMEX Sp. z o. o., Szczucin, Poland) at the temperature recommended by the manufacturer (95–100 °C). The quality of water was measured with TDS (total dissolved solids) meter (TDS-3, ABC-RC, Wieprz, Poland). The TDS value was 7 mg/L. An electric kettle (Amica model KM6011, Wronki, Poland) was used to control the temperature of the water used to prepare tea infusions. The bags were brewed for three minutes under a cover to prevent evaporation. For loose-leaf teas, 2.0 g of tea was similarly infused with 200 mL of deionized water at the indicated temperature and brewed for three minutes under cover. To obtain a clear infusion, the tea was filtered through filter paper. For tea bags weighing less than 2.0 g (e.g., 1.4 g, 1.5 g, or 1.75 g), the results were normalized to a 2.0 g equivalent to match the mass of the loose-leaf samples. After cooling to room temperature, the infusions were subjected to laboratory analysis. Each of the examined parameters was measured in triplicate to ensure the reliability of the results.

### 2.3. Measuring the pH of Herbal Infusions

The pH was measured using a Eurosensor pH electrode (Eurosensor, Gliwice, Poland) connected to a CPI-505 pH/ion meter (Elmetron, Zabrze, Poland). All measurements were performed at room temperature using freshly prepared infusions. The reagents used were of analytical grade, and solutions were prepared with deionized water (TDS = 7 mg/L, EUROMEX Sp. z o. o., Szczucin, Poland).

### 2.4. Measuring the Buffering Capacity of Tea Infusions

Buffering capacity refers to a substance’s ability to resist changes in pH upon the addition of an acid or base. In this study, the buffering capacity was determined by adding 0.1 M HCL (reagent grade, Chempur, Poland) to the infusion and calculated using the following formula:

(1) $$Buffer\;capacity\;(mol/L)=(0.01/V)\times ({pH}_{2}-{pH}_{1})$$

where

• *pH*_1_ is the pH of the brewed tea;
• *pH*_2_ is the pH after the addition of 0.1 M HCl.

### 2.5. Determination of Titratable Acidity of Tea Infusions

Titratable acidity, expressed in millimoles per liter (mmol/L), represents the volume of 0.1 M NaOH (reagent grade, Chempur, Poland) needed to neutralize all acidic components in the solution to a pH of 7.

### 2.6. Determination of Inorganic Phosphorus and Calcium in Tea Infusions

The concentration of inorganic phosphorus was determined using the phosphomolybdate method at a wavelength of 340 nm, with deionized water as a reference (TDS = 7 mg/L, EUROMEX Sp. z o. o., Szczucin, Poland). The method is based on the formation of a phosphomolybdate complex in an acidic medium, followed by reduction to a molybdenum blue complex measurable spectrophotometrically. The reagents used were ammonium molybdate, sulphuric acid, and detergents (reagent grade, Spinreact Kit, Spinreact, S.A./S.A.U., Sant Esteve de Bas, Spain).

Calcium was determined using the Arsenazo III method at a wavelength of 630 nm, also with deionized water (TDS = 7 mg/L, EUROMEX Sp. z o. o., Szczucin, Poland) as a reference. The method is based on the formation of a stable purple complex between calcium ions and arsenazo III in imidazole buffer pH 6.5 (reagent grade, Spinreact Kit, Spinreact, S.A./S.A.U., Sant Esteve de Bas, Spain).

### 2.7. Determination of Fluoride Content in Tea Infusions

Fluoride was determined using an ORION 9609 ion-selective electrode (Thermo Fisher Scientific, Waltham, MA, USA), in accordance with the manufacturer’s instructions, in conjunction with a CPI-551 ELMETRON pH/ionometer microcomputer (Elmetron, Zabrze, Poland). This electrode is classified as a combined electrode, meaning that a separate reference electrode was not required.

### 2.8. Statistical Analysis

The statistical analysis performed in this study focused on identifying differences between tea types (bags, loose-leaf, and pyramid bags) and regions (Africa, Central Asia, and East Asia), as well as on finding correlations between the evaluated variables. Therefore, an ANOVA test with a Tukey post hoc test and a Pearson correlation test were used. Correlation analysis was performed for all evaluated teas, as well as for tea-type subgroups. The analysis was conducted using the Jamovi software v. 2.6 (Jamovi, Sydney, Australia) and R Statistical Environment (R Core Team, Vienna, Austria).

## 3. Results

### 3.1. Physicochemical Properties of Teas

**Figure 1:**
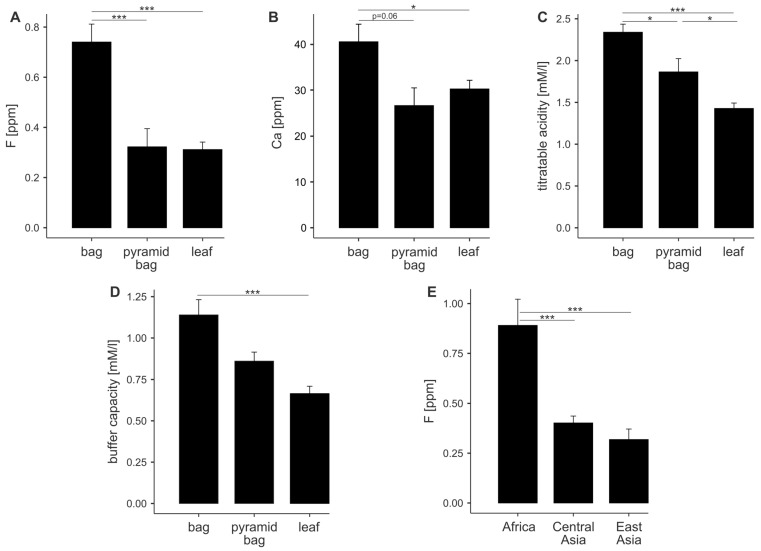
Selected physicochemical properties of black teas depending on form (leaf, bag, and pyramid bag) and region of origin. Bar plots (**A**–**D**) show statistically significant differences between different tea forms, while chart (**E**) presents differences based on origin. * *p* < 0.05, ; *** *p* < 0.001.

Statistically significant differences in the analyzed physicochemical properties are presented in Figure 1. The effect of tea form on the results is shown in Figure 1A–D. Tea bags exhibit the highest levels of fluoride, calcium, titratable acidity, and buffer capacity. Teas in pyramid bags and loose-leaf form tend to show similar values, except for titratable acidity, where loose-leaf tea displays the lowest value. The raw descriptive values for the different groups are included in Supplementary Table S1.

Teas produced in different regions exhibit similar characteristics. The only statistically significant difference was observed in fluoride concentration, with teas from Africa containing approximately twice as much fluoride as those from Central or East Asia (Figure 1E).

### 3.2. Correlation Analysis Among Physicochemical Properties

**Figure 2:**
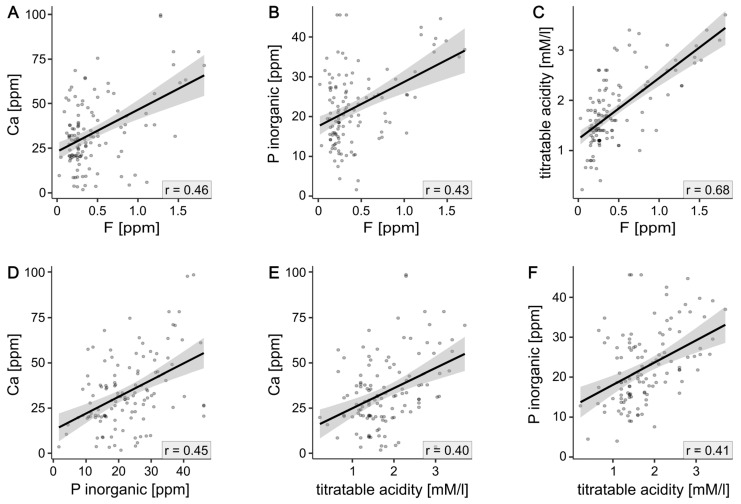
Correlation analysis of selected physicochemical properties. Each dot refers to a distinct tea type. Black lines show overall trend. (**A**) correlation plot for F and Ca. (**B**) inorganic P and F. (**C**) titratable acidity and F. (**D**) Ca and inorganic P. (**E**) titratable acidity and Ca. (**F**) inorganic P and titratable adicity.

Statistically significant correlations are presented in Figure 2. Correlation analysis was conducted for all evaluated teas, as well as within specific tea-type subgroups. Overall, the concentrations of all analyzed elements showed significant mutual correlations, as follows: fluoride with calcium (r = 0.46), fluoride with inorganic phosphorus (r = 0.43), and inorganic phosphorus with calcium (r = 0.45). Additionally, all three elements correlated significantly with titratable acidity (fluoride: r = 0.68, calcium: r = 0.40, and phosphorus: r = 0.41).

Notably, when the correlations were analyzed within individual subgroups, they remained significant only for tea-bag infusions. In loose-leaf teas, the correlations between calcium and fluoride, as well as phosphorus and fluoride, were not statistically significant. Tea in pyramid bags showed a significant correlation only between fluoride and titratable acidity.

## 4. Discussion

The present study demonstrates that the physical form and processing method of black tea significantly affects fluoride content in brewed infusions. The results indicate that tea bags showed the highest fluoride concentrations compared with loose-leaf and pyramid bags, consistent with previous observations [1,4] that a smaller particle size and increased surface area increase fluoride extraction during brewing [8,25]. Additionally, the quality grading system often assigns lower-grade leaves with potentially higher fluoride accumulation to tea bags, while premium whole leaves are reserved for loose-leaf products [11,26]. This increased extraction efficiency in tea bags is concerning from a public health perspective, as tea bags are the most widely consumed form of black tea in the world. The strong correlations between fluoride and other minerals (calcium r = 0.46 and inorganic phosphorus r = 0.43) observed exclusively in tea bags suggest that the processing and packaging methods significantly alter the chemical matrix and release characteristics of bioactive compounds. These findings extend beyond previous studies that focused primarily on brewing parameters, demonstrating that the commercial form of tea itself is a critical determinant of fluoride exposure, with bag varieties potentially contributing disproportionately to total dietary fluoride intake.

The significant regional variation in fluoride content observed in this study, particularly the elevated levels in African-sourced teas compared with Central and East Asian varieties, reflects the complex interaction between environmental factors and tea plant fluoride bioaccumulation, confirming previous observations by Pavlovič et al. regarding geographic variability in tea fluoride content [25]. For populations in areas with fluoridated water supplies, the cumulative effect of tea consumption could easily exceed safe fluoride thresholds, especially in children, whose lower body weight makes them more susceptible to fluoride toxicity [27,36]. These findings support the need for evidence-based consumption guidelines that consider tea form, origin, and individual risk factors [36]. Our results indicating approximately twice the fluoride concentrations in African teas support previous studies showing that *Camellia sinensis* acts as a bioaccumulator and that fluoride uptake depends on soil composition, water quality, and agricultural practices specific to growing regions [28,29,30]. This geographic variability has major implications for dietary risk assessment, as consumers of teas from Africa may experience significantly higher fluoride exposure than those consuming teas from other regions. The observed bioaccumulation patterns are consistent with established knowledge that mature tea leaves concentrate fluoride over time, but our findings uniquely quantify the regional discrepancies that exist in commercial tea products. Public health recommendations should include fluoride labeling on tea products, especially for high-risk varieties, and educational initiatives to promote awareness of cumulative fluoride exposure from all dietary sources, as suggested by Satou et al. in their risk assessment framework [31].

The clinical significance of our findings lies in the potential impact of chronic tea consumption on fluoride-related health outcomes, both beneficial and adverse. While moderate fluoride intake provides well-documented prevention of dental caries through enamel remineralization and antimicrobial activity against caries-forming bacteria [32,33,34,41], our results indicate that consumption at the level of 3–4 cups per day, especially of high-fluoride varieties, may result in fluoride intake approaching or exceeding recommended thresholds, especially when combined with other fluoride sources. The link between fluoride and health outcomes suggests that elevated concentrations observed in certain forms and origins of tea may contribute to dental fluorosis in children and, with prolonged exposure, potentially to skeletal fluorosis [19,42,43]. However, the antioxidant, anti-inflammatory, and cardiovascular benefits of black tea compounds such as theaflavins and thearubigins should be weighed against fluoride risks in clinical decision making. Our results support the implementation of individualized dietary counseling that takes into account total fluoride exposure from all sources, including tea consumption patterns, particularly for patients at high risk for fluoride-related adverse effects. This personalized approach is consistent with contemporary principles of preventive medicine and the growing recognition that dietary interventions must address cumulative patterns of exposure rather than isolated dietary components.

The findings of this study underscore the potential of black tea—especially in bag form—as a significant and often underestimated source of dietary fluoride, with clear implications for public health. Infusion of tea bags exhibited consistently higher fluoride concentrations than infusion of loose-leaf and pyramid bags, likely due to differences in physical form and infusion efficiency. Notably, only tea-bag infusions demonstrated strong correlations between fluoride and other elements, indicating their distinct role in cumulative fluoride exposure. These distinctions are especially salient considering that regular consumption of black tea, estimated at 1–1.5 L per day, may contribute 50–100% of the AI, and in some instances may approach or exceed UL, particularly in populations with additional exposure from fluoridated water or dental products [44]. The substantial regional variability observed—especially the elevated fluoride levels in African-sourced teas—further emphasizes the need for individualized risk assessment. These findings align with current recommendations from global and national health authorities, including the WHO, EFSA, and Polish expert bodies, and contribute novel and form- and origin-specific data essential for refining dietary guidelines and optimizing preventive strategies against fluoride-related health risks [45,46].

The infusion water used is a critical factor in determining the overall fluoride content of the beverage. The final fluoride content of the tea extract is influenced by water-related factors, including water hardness, calcium and fluoride content, and pH. Our experience from previous studies [47] shows that tap water varies greatly in terms of the aforementioned parameters, even within a single city. For instance, in the city of Wrocław (Poland), the calcium content of the water measured at three water production stations (‘Na Grobli’, ‘Mokry Dwór’, and ‘Leśnica—pumping station II degree’) was found to be within the range of 0.0860–0.1490 mg/mL. Furthermore, the presence of a range of physico-chemical parameters in commercially available bottled water samples on the Polish market is likely to result in fluctuations in fluoride levels in the beverage. The utilization of deionized water in our experimental protocol was intended to provide an objective assessment of the fluoride content in extracts obtained from individual teas. It is important to note that these conditions are essential for ensuring the reliability of the measurement process. Consequently, the fluoride content of the tea sample is the only source of variation in the results.

The aluminum (Al^3+^) present in the extracts demonstrates a pronounced propensity to form complexes with fluoride (Al-F), particularly in acidic environments, where it is present as soluble ions. This results in a reduction in the concentration of free fluoride ions (F-) in solution. In the Al^3+^/F- system, forms such as AlF^2+^, AlF_2_^+^, AlF^30^, AlF_4_^−^, AlF_5_^2−^, and AlF_6_^3−^ occur, and their distribution depends on pH, among other factors [48,49,50]. In the context of scientific studies, it has been demonstrated that within an environment characterized by a pH value of approximately 4–6, a substantial proportion of fluoride, estimated to be over 78%, is found to be complexed with aluminum. This observation indicates that a mere 21% of the fluoride remains in its free state [51,52]. The addition of milk or sugar to infusions by consumers has been demonstrated to influence the fluoride delivery with the beverage. The high calcium content of milk has been shown to reduce the bioavailability of fluoride by approximately 20–80% through the formation of insoluble CaF_2_ in the gastrointestinal tract, thereby reducing the absorption of this element [53,54]. Conversely, the addition of sugar has been observed to enhance the extraction of fluoride during the initial minutes of tea steeping [55]. It is, therefore, postulated that knowledge of the aforementioned interactions may serve to lower the threshold for potentially harmful fluoride intake.

While this study offers valuable insights into the fluoride content of black tea infusions across different forms and regions, several limitations should be acknowledged. The analysis was limited to teas available in a single market (Poland), which may affect the generalizability of the findings to other regions with different consumption patterns and product types. Although fluoride levels were measured under standardized conditions, real-life variables—such as water fluoride content, brewing duration, and temperature—may significantly alter actual exposure. The study also did not account for individual variability in fluoride absorption and metabolism, which can influence systemic fluoride burden. As a cross-sectional analysis, it cannot inform long-term health outcomes. Future research should include a wider geographic scope, more diverse brewing scenarios, and a comprehensive assessment of all fluoride sources. Longitudinal studies are particularly needed to assess the cumulative health effects of fluoride from tea, with particular attention paid to children who are most susceptible to dental fluorosis due to their greater sensitivity to fluoride during tooth development.

## 5. Conclusions

Black tea, particularly in bag form and sourced from Africa, is a notable contributor to daily fluoride intake. With regular consumption, especially alongside other fluoride sources, like drinking water or dental products, there is potential to exceed recommended intake levels—posing a risk, particularly for children. The study highlights that both tea form and origin significantly influence fluoride content, with tea bags showing the highest concentrations and strongest mineral correlations. These findings support the need for clearer labeling of fluoride content on tea products and greater consumer awareness. Public health recommendations should account for individual consumption habits and regional product differences to better manage fluoride exposure and its associated health risks.

## Figures and Tables

**Table 1:** Types of teas included into the study.

Tea Name (Brand)	Form	Origin
Assam Irish Breakfast (Ronnefeldt)	Bags	India
Classic Black Tea (Tetley)	Bags	India
Classic Earl Grey (Tetley)	Bags	India
Darjeeling (Ronnefeldt)	Bags	India
Earl Grey (Ronnefeldt)	Bags	India
Yellow Label (Lipton)	Bags	India
Black with Ginger and Turmeric (Remsey)	Bags	Kenya
Classic Black (Remsey)	Bags	Kenya
Earl Grey (Lord Nelson)	Bags	Kenya
Earl Grey Strong Tempting Bergamot Flavour (Remsey)	Bags	Kenya
Earl Grey with a Hint of Lemon Bergamot Flavour (Remsey)	Bags	Kenya
English Breakfast (Lipton)	Bags	Kenya
English Teatime (Lipton)	Bags	Kenya
Yellow Label (Lipton)	Bags	Kenya
Classic Black (Lord Nelson)	Bags	Outside EU
Black Tea “Minutka” (Minutka)	Bags	Sri Lanka
Black Tea (Minutka)	Bags	Sri Lanka
Black Tea Earl Grey Full-Bodied (Vintage Teas)	Bags	Sri Lanka
Ceylon (Loyd)	Bags	Sri Lanka
Ceylon Black (Remsey)	Bags	Sri Lanka
Ceylon Black Tea (Big-Active)	Bags	Sri Lanka
Ceylon Earl Grey (Remsey)	Bags	Sri Lanka
Ceylon Gold (Sir William’s Tea)	Bags	Sri Lanka
Ceylon Premium Tea (Dilmah)	Bags	Sri Lanka
Classic Earl Grey (Exclusive) (Lipton)	Bags	Sri Lanka
Earl Grey (Ahmad Tea)	Bags	Sri Lanka
Earl Grey (Akbar)	Bags	Sri Lanka
Earl Grey (Loyd)	Bags	Sri Lanka
Earl Grey (Loyd)	Bags	Sri Lanka
Earl Grey (Sir William’s Tea)	Bags	Sri Lanka
Earl Grey (Teekane)	Bags	Sri Lanka
English Breakfast (Ahmad Tea)	Bags	Sri Lanka
English Breakfast (Ronnefeldt)	Bags	Sri Lanka
English Breakfast (Sir William’s Tea)	Bags	Sri Lanka
English Breakfast Tea (Remsey)	Bags	Sri Lanka
English Tea No. 1 (Ahmad Tea)	Bags	Sri Lanka
Finest Black Tea (Eternal)	Bags	Sri Lanka
Gold (Tekkane)	Bags	Sri Lanka
Gold Tea (Loyd)	Bags	Sri Lanka
Intense Black (Lipton)	Bags	Sri Lanka
Lemon Black (Minutka)	Bags	Sri Lanka
Royal Elixir Tea (Impra Tea)	Bags	Sri Lanka
Eternal Finest (Eternal)	Granulate	India
Golden Assam (Golden Assam)	Granulate	India
Yellow Label (Lipton)	Granulate	Kenya
Black Tea (Yunnan)	Leaves	China
China Black Golden Silk Missing (Five o’clock)	Leaves	China
China Panyong Golden Needle (Five o’clock)	Leaves	China
Earl Grey with Lemon Zest (Lord Nelson)	Leaves	China
Formosa Lapsang Souchong (Five o’clock)	Leaves	China
Golden Yunan (Five o’clock)	Leaves	China
Golden Yunan Superior (Five o’clock)	Leaves	China
Keemun (Five o’clock)	Leaves	China
Yunan Black Tea (ZAS)	Leaves	China
Yunnan (Loyd)	Leaves	China
Yunnan (ZAS)	Leaves	China
Georgian Black Wild Op (Five o’clock)	Leaves	Georgia
Assam (Lord Nelson)	Leaves	India
Assam (ZAS)	Leaves	India
Assam Halmari GTGFBOP (Five o’clock)	Leaves	India
Assam Halmari GTGFOP1CL (Five o’clock)	Leaves	India
Assam Satrupa (Five o’clock)	Leaves	India
Assam Singlijan (Five o’clock)	Leaves	India
Assam Tea (Ahmad Tea)	Leaves	India
Assam Tonganagaon (Five o’clock)	Leaves	India
Ceylon Lumbini (Five o’clock)	Leaves	India
Darjeeling Flower Balasun (Five o’clock)	Leaves	India
Darjeeling Gielle (Five o’clock)	Leaves	India
Darjeeling Liza Hill DJ5/21 (Five o’clock)	Leaves	India
Darjeeling Musk Puttabang (Five o’clock)	Leaves	India
Darjeeling Nagr DJ2 (Five o’clock)	Leaves	India
Darjeeling Shree Dwarika (Five o’clock)	Leaves	India
Darjeeling Teesta Valley DJ11 (Five o’clock)	Leaves	India
Madras (Loyd)	Leaves	India
Madras (ZAS)	Leaves	India
Pure Ceylon Tea (Akbar)	Leaves	India
South India Nilgiri Kukicha Roasted (Five o’clock)	Leaves	India
Japan Black Tea (Five o’clock)	Leaves	Japan
Safari (Astra)	Leaves	Kenya
Yellow Label (Lipton)	Leaves	Kenya
Laos Black Saylom (Five o’clock)	Leaves	Laos
Nepal Arubote (Five o’clock)	Leaves	Nepal
Nepal Yun Chiyabari Himalayan Imperial (Five o’clock)	Leaves	Nepal
Kivu Lake Selected (HAYB)	Leaves	Rwanda
Rwanda Rukei Op (Five o’clock)	Leaves	Rwanda
Black Tropical Tea (Sir Adalbert’s Tea)	Leaves	Sri Lanka
Ceylon (Big-Active)	Leaves	Sri Lanka
Ceylon Ahinsa (Five o’clock)	Leaves	Sri Lanka
Ceylon Gold (Dilmah)	Leaves	Sri Lanka
Ceylon Premium Tea (Dilmah)	Leaves	Sri Lanka
Ceylon Supreme (Dilmah)	Leaves	Sri Lanka
Ceylon Tea (Ahmad Tea)	Leaves	Sri Lanka
Ceylon Vithanakande (Five o’clock)	Leaves	Sri Lanka
Dimbula (Vintage Teas)	Leaves	Sri Lanka
Earl Grey (Ahmad Tea)	Leaves	Sri Lanka
Earl Grey (Akbar)	Leaves	Sri Lanka
Earl Grey (Loyd)	Leaves	Sri Lanka
Earl Grey (Sir Adalbert’s Tea)	Leaves	Sri Lanka
Earl Grey (Vintage Teas)	Leaves	Sri Lanka
English Breakfast (Ahmad Tea)	Leaves	Sri Lanka
English Breakfast (Dilmah	Leaves	Sri Lanka
English Royal Tea (Chelton)	Leaves	Sri Lanka
English Tea No. 1 (Ahmad Tea)	Leaves	Sri Lanka
Finest Top (Eternal)	Leaves	Sri Lanka
Gourmet Earl Grey Tea (Dilmah)	Leaves	Sri Lanka
Kandy (Vintage Teas)	Leaves	Sri Lanka
Nuwara Eliya (Vintage Teas)	Leaves	Sri Lanka
Organic Black Tea (Vintage Teas)	Leaves	Sri Lanka
Royal Elixir Tea (Gold) (Impra Tea)	Leaves	Sri Lanka
Ruhuna (Vintage Teas)	Leaves	Sri Lanka
Sabaragamuwa (Vintage Teas)	Leaves	Sri Lanka
Darjeeling Tea (Teapigs)	Pyramid bags	India
English Breakfast (Teapigs)	Pyramid bags	India
Earl Grey (English Teashop Organic)	Pyramid bags	Sri Lanka
Earl Grey (Vintage Teas)	Pyramid bags	Sri Lanka
Earl Grey Strong (Teapigs)	Pyramid bags	Sri Lanka
Elegant Earl Grey (Dilmah)	Pyramid bags	Sri Lanka
English Breakfast (Dilmah)	Pyramid bags	Sri Lanka
English Breakfast (English Teashop Organic)	Pyramid bags	Sri Lanka
English Breakfast (Vintage Teas)	Pyramid bags	Sri Lanka
Maharaja Reserve Assam (Dilmah)	Pyramid bags	Sri Lanka
Organic Black Tea Broken Orange Pekoe KANDY (Vintage Teas)	Pyramid bags	Sri Lanka
Perfect Ceylon Tea (Dilmah)	Pyramid bags	Sri Lanka
Valley of Kings Ceylon Pekoe (Dilmah)	Pyramid bags	Sri Lanka

## Data Availability

The original contributions presented in this study are included in the article/Supplementary Materials. Further inquiries can be directed to the corresponding authors.

## Supplementary Materials

The following supporting information can be downloaded at: https://www.mdpi.com/article/10.3390/foods14142452/s1, Table S1: Raw descriptive statistics.
