# Supplementary material for: Fluoride Content in Infusions of Selected Teas Available on the Polish Market—An In Vitro Study
Source: Foods. 2025 Jul 12;14(14):2452. doi: 10.3390/foods14142452 (PMC12294286; doi:10.3390/foods14142452)
Supplement: Supplementary file 1 [file foods-14-02452-s001.zip › foods-3724713-supplementary.pdf]

Table S1. Raw descriptive statistics.

Variable	N	Mean	Median	Minimum	Maximum	Stand. Deviation
**ALL TEAS**						
**pH**	121	4.71	4.64	4.33	5.70	0.26
**F [ppm]**	121	0.46	0.31	0.03	1.82	0.39
**buffer capacity [mM/l]**	121	0.85	0.71	0.09	2.50	0.49
**titratable acidity [mM/l]**	121	1.79	1.64	0.22	3.70	0.70
**Ca [ppm]**	121	33.46	30.86	1.64	98.60	19.62
**P inorganic [ppm]**	120	22.39	20.98	1.70	45.53	9.46
**REGIONS**						
**Africa**						
**pH**	16	4.66	4.65	4.54	4.91	0.09
**F [ppm]**	16	0.89	0.78	0.22	1.75	0.53
**buffer capacity [mM/l]**	16	1.03	0.85	0.09	2.50	0.71
**titratable acidity [mM/l]**	16	2.24	2.25	1.00	3.40	0.79
**Ca [ppm]**	16	38.95	31.26	8.90	78.16	22.11
**P inorganic [ppm]**	16	26.63	24.78	10.28	44.60	9.98
**Central Asia**						
**pH**	81	4.73	4.64	4.42	5.70	0.29
**F [ppm]**	81	0.40	0.28	0.03	1.44	0.31
**buffer capacity [mM/l]**	81	0.87	0.77	0.11	2.50	0.47
**titratable acidity [mM/l]**	81	1.75	1.68	0.22	3.33	0.64
**Ca [ppm]**	81	31.66	29.66	1.64	98.60	19.01
**P inorganic [ppm]**	80	22.06	21.68	1.70	45.53	9.31
**East Asia**						
**pH**	14	4.63	4.59	4.33	5.22	0.21
**F [ppm]**	14	0.32	0.26	0.15	0.84	0.20
**buffer capacity [mM/l]**	14	0.77	0.64	0.31	1.67	0.37
**titratable acidity [mM/l]**	14	1.64	1.40	1.10	3.40	0.67
**Ca [ppm]**	14	34.87	36.87	8.42	67.73	18.04
**P inorganic [ppm]**	14	19.2	17.03	8.98	29.42	6.81
**Other/unambiguous**						
**pH**	10	4.73	4.62	4.41	5.24	0.25
**F [ppm]**	10	0.46	0.24	0.11	1.82	0.53
**buffer capacity [mM/l]**	10	0.56	0.49	0.24	1.27	0.29
**titratable acidity [mM/l]**	10	1.57	1.50	0.64	3.70	0.89
**Ca [ppm]**	10	37.27	38.88	4.41	70.54	22.84
**P inorganic [ppm]**	10	22.92	18.27	10.53	45.53	11.77
**TEA TYPES**						
**Leaf**						
**pH**	66	4.74	4.63	4.33	5.67	0.31
**F [ppm]**	66	0.31	0.26	0.05	1.52	0.24
**buffer capacity [mM/l]**	66	0.66	0.55	0.11	2.00	0.36
**titratable acidity [mM/l]**	66	1.43	1.40	0.22	3.40	0.53
**Ca [ppm]**	66	30.28	27.25	3.25	67.74	15.31
**P inorganic [ppm]**	66	21.09	18.58	3.96	45.53	9.31
**Pyramid bags**						

**pH**	13	4.62	4.59	4.42	5.00	0.15
**F [ppm]**	13	0.32	0.25	0.03	1.10	0.26
**buffer capacity [mM/l]**	13	0.86	0.80	0.63	1.25	0.20
**titratable acidity [mM/l]**	13	1.86	1.64	1.36	3.10	0.57
**Ca [ppm]**	13	26.65	31.26	1.64	43.89	13.92
**P inorganic [ppm]**	13	20.33	20.47	8.92	30.51	6.99
**Bag tea**						
**pH**	42	4.70	4.65	4.48	5.70	0.20
**F [ppm]**	42	0.74	0.56	0.15	1.82	0.46
**buffer capacity [mM/l]**	42	1.14	0.97	0.09	2.50	0.60
**titratable acidity [mM/l]**	42	2.34	2.30	0.40	3.70	0.62
**Ca [ppm]**	42	40.56	37.43	3.49	98.60	24.81
**P inorganic [ppm]**	41	25.15	24.78	1.70	42.43	9.92
